# Can Cyanobacterial Diversity in the Source Predict the Diversity in Sludge and the Risk of Toxin Release in a Drinking Water Treatment Plant?

**DOI:** 10.3390/toxins13010025

**Published:** 2021-01-01

**Authors:** Farhad Jalili, Hana Trigui, Juan Francisco Guerra Maldonado, Sarah Dorner, Arash Zamyadi, B. Jesse Shapiro, Yves Terrat, Nathalie Fortin, Sébastien Sauvé, Michèle Prévost

**Affiliations:** 1 Department of Civil, Geological and Mining Engineering, Polytechnique Montréal, Montréal, QC H3C 3A7, Canada; hana.trigui@polymtl.ca (H.T.); juan-francisco.guerra-maldonado@polymtl.ca (J.F.G.M.); sarah.dorner@polymtl.ca (S.D.); michele.prevost@polymtl.ca (M.P.); 2 Water Research Australia, Adelaide SA 5001, Australia; arash.zamyadi@waterra.com.au; 3 Department of Biological Sciences, University of Montréal, Montréal, QC H2V 0B3, Canada; jesse.shapiro@umontreal.ca (B.J.S.); yves.terrat@umontreal.ca (Y.T.); 4 Department of Microbiology and Immunology, McGill University, Montréal, QC H3A 2B4, Canada; 5 McGill Genome Center, McGill University, Montréal, QC H3A 0G1, Canada; 6 National Research Council Canada, Energy, Mining and Environment, Montréal, QC H4P 2R2, Canada; nathalie.fortin@cnrc-nrc.gc.ca; 7 Department of Chemistry, University of Montréal, Montréal, QC H3C 3J7, Canada; sebastien.sauve@umontreal.ca

**Keywords:** cyanobacteria, microcystins (MCs), water treatment, sludge, shotgun metagenomics, cyanobacterial community, high-throughput sequencing

## Abstract

Conventional processes (coagulation, flocculation, sedimentation, and filtration) are widely used in drinking water treatment plants and are considered a good treatment strategy to eliminate cyanobacterial cells and cell-bound cyanotoxins. The diversity of cyanobacteria was investigated using taxonomic cell counts and shotgun metagenomics over two seasons in a drinking water treatment plant before, during, and after the bloom. Changes in the community structure over time at the phylum, genus, and species levels were monitored in samples retrieved from raw water (RW), sludge in the holding tank (ST), and sludge supernatant (SST). *Aphanothece clathrata brevis, Microcystis aeruginosa, Dolichospermum spiroides,* and *Chroococcus minimus* were predominant species detected in RW by taxonomic cell counts. Shotgun metagenomics revealed that Proteobacteria was the predominant phylum in RW before and after the cyanobacterial bloom. Taxonomic cell counts and shotgun metagenomic showed that the *Dolichospermum* bloom occurred inside the plant. Cyanobacteria and Bacteroidetes were the major bacterial phyla during the bloom. Shotgun metagenomics also showed that *Synechococcus*, *Microcystis,* and *Dolichospermum* were the predominant detected cyanobacterial genera in the samples. Conventional treatment removed more than 92% of cyanobacterial cells but led to cell accumulation in the sludge up to 31 times more than in the RW influx. Coagulation/sedimentation selectively removed more than 96% of *Microcystis* and *Dolichospermum*. Cyanobacterial community in the sludge varied from raw water to sludge during sludge storage (1–13 days). This variation was due to the selective removal of coagulation/sedimentation as well as the accumulation of captured cells over the period of storage time. However, the prediction of the cyanobacterial community composition in the SST remained a challenge. Among nutrient parameters, orthophosphate availability was related to community profile in RW samples, whereas communities in ST were influenced by total nitrogen, Kjeldahl nitrogen (N- Kjeldahl), total and particulate phosphorous, and total organic carbon (TOC). No trend was observed on the impact of nutrients on SST communities. This study profiled new health-related, environmental, and technical challenges for the production of drinking water due to the complex fate of cyanobacteria in cyanobacteria-laden sludge and supernatant.

## 1. Introduction

Cyanobacterial cells and their associated cyanotoxins are considered to represent an important challenge due to (1) their health threat to humans and animals; (2) their negative aesthetic impacts with respect to taste, odor, and color; (3) the implications of extra water treatment requirements for ozonation and membrane filtration; and (4) the increased consumption of coagulants, flocculants, and activated carbon [1,2,3].

Conventional treatment using coagulation, flocculation, sedimentation, and filtration is a common approach for cyanobacterial removal from intake water [1,3,4]. However, conventional treatment is not efficient at removing dissolved cyanotoxins [5]. In addition, hydraulic and chemical stresses during treatment may cause damage to cells and trichomes, leading to the release of cyanotoxins [6,7]. Another challenge of conventional treatment is the increase in cyanobacteria and cyanotoxin concentrations in the clarifiers and filters of water treatment plants (WTPs) and their accumulation in the sludge of clarifiers [3,4,8,9]. Cyanobacterial cells can be present at concentrations 10–100 times higher in the sludge than in intake water, even in plants with low cyanobacterial flux (<1000 cells/mL) [10,11]. Moreover, some investigations have shown that coagulated cells can stay viable in the sludge for 2 to 10 days [4,9,12,13,14]. More recently, cell viability in the sludge was observed for more than 20 days [15]. During this period, microcystin-LR (MCLR) and cylindrospermopsin concentrations increased to 3–7 times their initial levels. The authors of [16] reported that metabolite concentrations in the sludge supernatant after storage were up to five times greater than those within the sludge before storage. This shows a new challenge in sludge management during storage and when the sludge supernatant is recycled to the head of the plant [16,17]. The fate of cyanobacteria and cyanotoxins during and after coagulation and in the sludge is not fully understood. The impacts of coagulation on cyanobacterial cells are still controversial. Although some studies have demonstrated that coagulation depends on cyanobacterial species [3,17,18], another study showed that cells are not selectively captured by coagulation [15]. It has been shown that cell damage and metabolites released in sludge are associated with various environmental conditions; however, due to the complex interactions of cyanobacteria with treatment processes, the primary factors behind this complex behavior are still not determined [16,19]. Additionally, although the positive impact of powdered activated carbon (PAC) on cyanotoxin degradation in raw water (RW) has been widely studied [5,20,21,22,23], there are no data about the role of injected PAC in RW in the degradation of accumulated cyanotoxins within sludge. 

Recently, high-throughput sequencing and metagenomics techniques have been successfully applied to describe microbial communities in the water resources to predict the occurrence of cyanobacterial blooms [24,25]. During the last decade, several studies have investigated bacterial communities in WTPs and have demonstrated that while microbial communities in the water treatment chain are represented by water intake, treatment processes have an impact on the microbial community structure through WTPs [17,26,27,28,29,30,31,32]. Few studies have investigated bacterial communities within sludge in WTPs [33,34]. The authors of [33] reported similar bacterial communities in sludge samples collected from six different Chinese WTPs with the same treatment processes. They reported similar bacterial communities in sludge samples, suggesting that bacterial communities in sludge might be shaped by RW communities; however, they did not compare the bacterial composition in sludge with that in RW. The authors of [34] studied the impact of different coagulants on bacterial communities and metabolite release in sludge. They found that the relative abundance of the dominant genera *Microcystis, Rhodobacter, Phenylobacterium,* and *Hydrogenophaga* decreased, reflecting their damage and the subsequent release of extracellular microcystin and organic matter. They suggest that the sludge should be treated or disposed of within 4 days to avoid the proliferation of the pathogens. 

There are basically no studies exploring the impact of RW cyanobacterial communities on cyanobacteria-laden sludge and its supernatant in WTPs. Moreover, previous studies considered sludge as a batch samples, while in WTPs cyanobacteria-laden sludge might be dynamically affected by different parameters such as RW characteristics, treatment process functionality, and sludge storage time. The impact of these parameters on the cyanobacterial community structure of sludge has not been investigated. No comparative analysis has been carried out on bacterial/cyanobacterial community composition within RW, sludge, and sludge supernatant. Due to the knowledge gaps related to the fate of cyanobacteria and cyanotoxins in sludge, high-throughput sequencing techniques could be useful to better understand the community dynamics of cyanobacteria-laden sludge through a WTP. This study is the first to use both shotgun metagenomic sequencing and taxonomic cell count approaches to provide an overview of cyanobacterial composition in RW, a sludge holding tank (ST), and the corresponding sludge supernatant (SST) in a WTP. 

The general objective of this research was to study the fate of cyanobacteria and its associated cyanotoxins in a WTP. The specific objectives were to: (1) diagnose critical points of WTPs where cyanobacteria cells and their associated cyanotoxins accumulate; (2) determine the relationship between cyanobacterial communities in RW, sludge, and its supernatant; (3) determine the impact of nutrients on cyanobacterial community shifts in RW, the sludge holding tank, and its supernatant; and (4) compare taxonomic cell counts with shotgun metagenomic sequencing results.

## 2. Results and Discussion

### 2.1. Impact of Conventional Treatment on Cyanobacteria and Cyanotoxins

**Figure 1:**
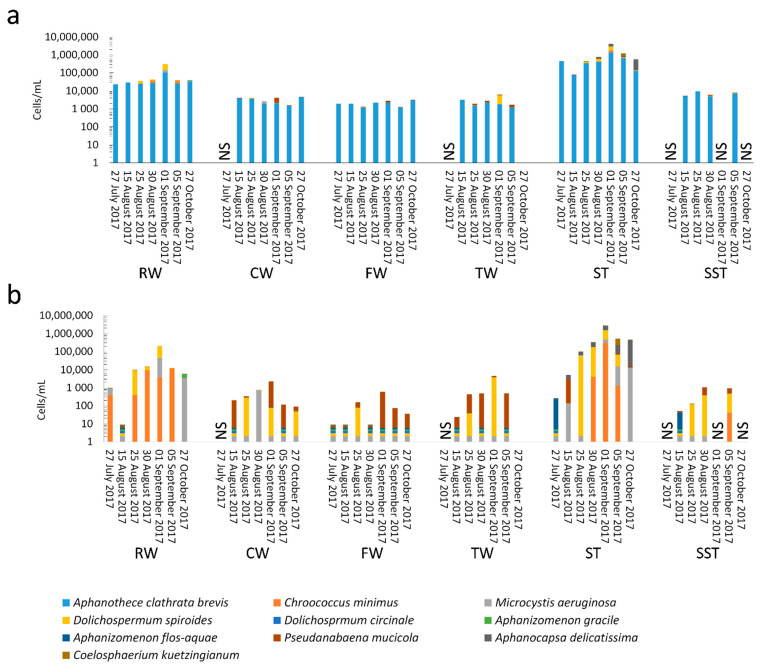
Distribution of cyanobacterial species in the water treatment plant (WTP) by taxonomic cell counts: (**a**) all species, (**b**) speciation of species other than *Aphanothece clathrata brevis*. RW: raw water, CW: clarified water, FW: filtered water, TW: treated water, ST: sludge holding tank, SST: sludge holding tank supernatant. Sludge storage times—27 July 2017: 8 days; 15 August 2017: 3 days; 25 August 2017: 8 days; 30 August 2017: 13 days; 1, 5 September 2017: 1 day; 27 October 2017: 2 days. NS: sample not taken.

**Figure 2:**
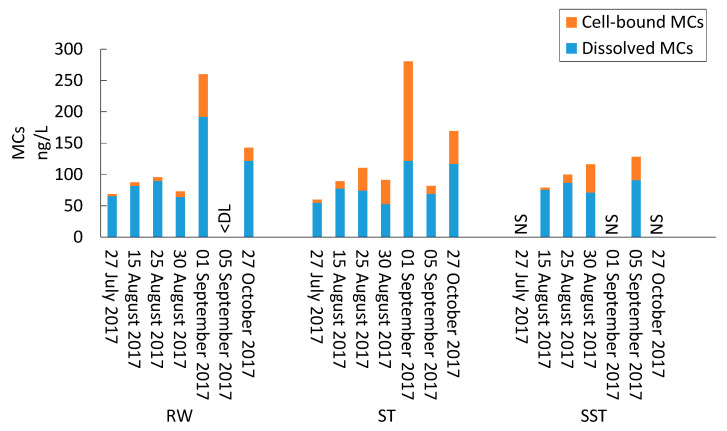
Concentration of dissolved and cell-bound microcystins (MCs) in raw water (RW), in the sludge holding tank (ST), and in sludge holding tank supernatant (SST). NS: sample not taken, DL: below detection limit.

During the period prior to the bloom (1 July to 30 August 2017), taxonomic cell counts in RW were below 5.0 × 10^4^ cells/mL and *Aphanothece clathrata brevis* was the most dominant species, representing 65–100% of total cell counts. *Dolichospermum spiroides* (0–26%), *Chroococcus minimus* (0–34%), *Microcystis aeruginosa* (0–2%), and *Dolichospermum circinale* (<1%) were detected frequently (Figure 1). During this period, low concentrations of MCs were detected, with dissolved microcystin (MC) levels below 90 ng/L and cell-bound MC levels below 10 ng/L (Figure 2, Table S2). 

A cyanobacterial bloom appeared in Missisquoi Bay in late August, and total taxonomic cell counts increased in RW to 3.1 × 10^5^ cells/mL on 1 September. The dominant species was *D. spiroides*, representing 52% of total cell counts with a concentration of 1.6 × 10^5^ cells/mL. Other identified species were *A. clathrata brevis* (1.0 × 10^5^ cells/mL, 33%), *M. aeruginosa* (4.0 × 10^4^ cells/mL, 13%), and *Coelosphaerium kuetzingianum* (1.7 × 10^3^ cells/mL, 1%) (Figure 1). The low total MC level increased to 260.1 ng/L, of which 191.9 ng/L were dissolved (Figure 2). On the sampling dates following the bloom (5 September and 27 October), cell counts decreased to around 3.9 × 10^4^ cells/mL and remained constant. The species *A. clathrata brevis* was dominant during those two dates (68–85%). *C. minimus* (31%) was also found on 5 September and *M. aeruginosa* (31%) and *Aphanizomenon gracile* (1%) were found on 27 October. MCs remained below the detection limit (DL) on 5 September. On 27 October, the total MC concentration increased to 142.6 ng/L, of which 121.9 ng/L were dissolved (Figure 2).

Through the treatment process, 86–99% of total cyanobacterial cells were removed by the clarifier. In particular, 85–100% of *M. aeruginosa*, *A. clathrata brevis*, *C. minimus, A. gracile,* and *D. spiroides* were eliminated. These results are in agreement with previous studies documenting coagulation efficiency of between 62% and 99% [4,8,14,35,36]. Meanwhile, 14–71% of the escaped cells from the clarifier were removed by filtration (Figure 1). Overall, 92–99% of cells were eliminated by conventional treatment. A previous study on this plant found a similar reduction [3]. Furthermore, taxonomic cell counts increased from 25% to 120% in treated water (TW) on all sampling dates. *A. clathrata brevis* (72–99%) remained dominant in TW, except on the bloom date (1 September). On 1 September, the cyanobacterial composition in TW consisted of *D. spiroides* (62%), *A. clathrata brevis* (29%), and *Pseudanabaena mucicola* (9%) (Figure 1). Cell counts in TW (after chlorination) were 1.3–2 times greater than cell counts in filtered water (FW) (before chlorination). This accumulation can be problematic if accumulated cells produce cyanotoxins.

Total taxonomic cell counts in ST remained around 3–31 times greater than in RW (Figure 1). The cell percentage of *A. clathrata brevis* in ST decreased from ~100% on 27 July to 32% on 1 September (bloom date). In contrast, during this period, the percentages of *Aphanocapsa delicatissima* and *D. spiroides* increased from 0% to 29% and 27%, respectively. On 1 September, *M. aeruginosa* counts in ST were four times greater than in RW. Furthermore, *A. delicatissima, P. mucicola, Aphanizomenon flos-aquae, D. circinale*, *and C. kuetzingianum* were detected in small amounts (0–1.7 × 10^3^ cells/mL) in RW, whereas their cell counts increased to between 2.5 × 10^2^ to 1.2 × 10^6^ cells/mL in ST on the corresponding dates (Figure 1). 

Cell counts in SST remained around 94–98% lower than those in ST and 69–97% lower than those in RW. *A. clathrata brevis* was dominant (83–99%) in SST, with small percentages of *D. spiroides* (1–6%) and *P. mucicola* (5–11%).

**Figure 3:**
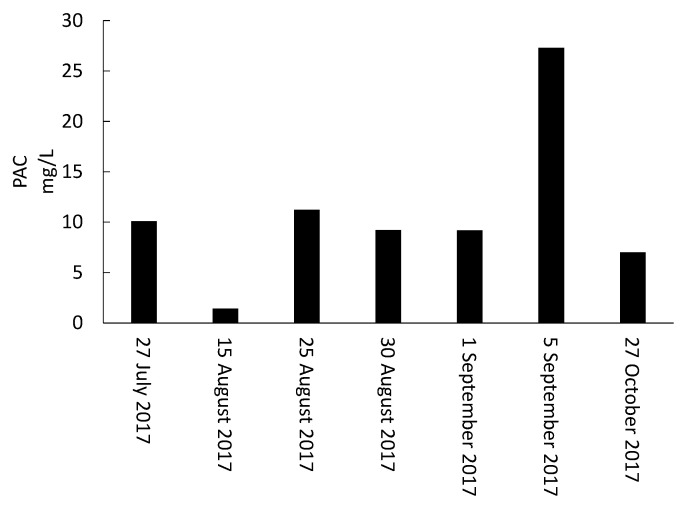
Powder activated carbon (wood-based PAC) doses injected into raw water (RW) during the 2017 sampling campaigns.

Total MCs in ST and SST remained below 281 and 128 ng/L, respectively, during the sampling campaign (Figure 2). These MC trends are inconsistent with previous investigations which reported MCLR concentrations in the clarifier sludge to be around 10 times greater than in RW [3,8]. This low concentration of MCs measured in the ST also contradicts the results of [16], where it was shown that cyanobacterial metabolites in lagoon supernatant were 2 to 5 times greater than the initial concentrations. One reason behind the low MC concentrations in the ST as well as in the SST in our study may be the impact of injected PAC in RW on accumulated MCs in stored sludge. Indeed, levels of dissolved MCs were reduced from 121.7 ng/L (1 September) to below DL on 5 September when the PAC dose increased from 9.2 to 27.3 mg/L. In contrast, the concentration of dissolved MCs increased to 116.7 ng/L on 27 October when the PAC dose decreased to 7.0 mg/L (Figure 2 and Figure 3). A second reason may be the biodegradation of MCs during sludge storage. However, the authors of [37] documented biodegradation of MCs as being very low compared to individual microcystin analogues.

### 2.2. Cyanobacterial Diversity in Sludge and Supernatant Assessed by Shotgun Metagenomic Sequencing 

**Figure 4:**
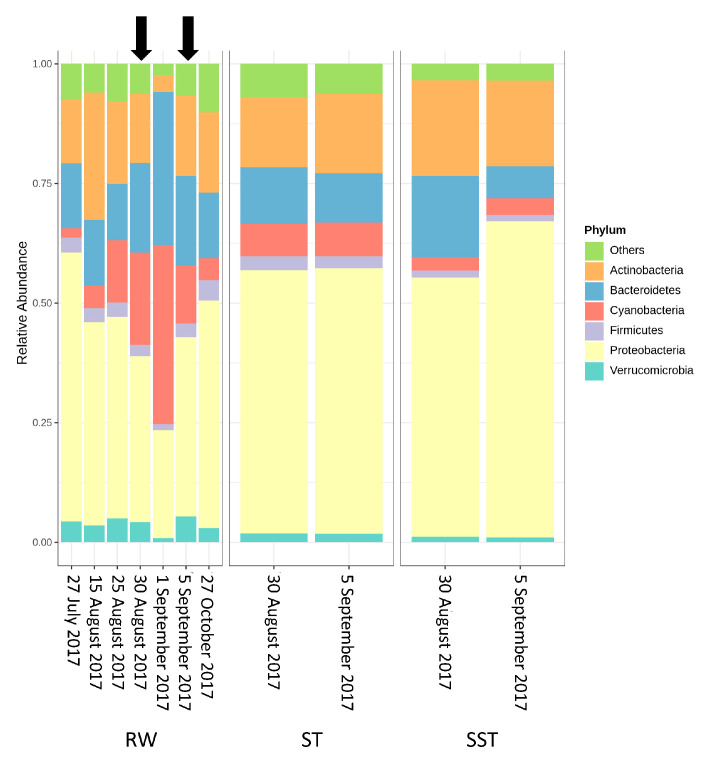
Bacterial community at the phylum level in raw water (RW) (27 July, 15, 25, and 30 August, 1 and 5 September, and 27 October 2017), in the sludge holding tank (ST), and in sludge holding tank supernatant (SST) (30 August, 5 September 2017). The black arrows show the corresponding dates with the ST and SST samples.

From 27 July to 25 August 2017, Proteobacteria remained dominant in RW and their relative abundance increased from 26% to 56%. Actinobacteria (12–26%) and Bacteroidetes (14%) were the following dominant phyla in RW. During this period, Cyanobacteria, Verrucomicrobia, and Firmicutes had small relative abundances below 6%, 5%, and 3%, respectively. On 30 August, the abundance of Proteobacteria and Actinobacteria decreased to 35% and 14.6%, respectively, while that of Cyanobacteria and Bacteroidetes increased to 19%. On 1 September, the community profile was associated with high cyanobacterial levels (38%) and was distinct from those of other sampling dates where there were lower cyanobacterial levels. This is coherent with trends observed in taxonomic cell count results on 1 September. Similarly, the relative abundance of Bacteroidetes reached its highest level (32%) on that date (Figure 4), as supported by previous reports linking cyanobacterial blooms with Bacteroidetes [38,39]. Indeed, Bacteroidetes is associated with nutrient loadings which promote the growth of Cyanobacteria [40]. In this work, we observed that total (TN) and dissolved nitrogen (DN) were significantly associated with the Bacteroidetes community (Table S1, Figure S1). On the next sampling dates (5 September and 27 October), the abundance of Cyanobacteria and Bacteroidetes decreased to 12.4% and 4.5% on 5 September, and 19% and 14% on 27 October, respectively. The abundance of Proteobacteria increased from 37% to 48% (Figure 4). On 30 August (before the bloom) and 5 September (after the bloom) Proteobacteria and Actinobacteria were the two dominant phyla in the ST (56–57% and 14–17%, respectively) and SST (56–68% and 18–20%, respectively) (Figure 4). During this period, the relative cyanobacterial abundance in the ST and SST was about 7% and 4%, respectively. Interestingly, Bacteroidetes was also found at low levels, fluctuating from 5% to 12% and from 7% to 18% in the ST and SST, respectively.

**Figure 5:**
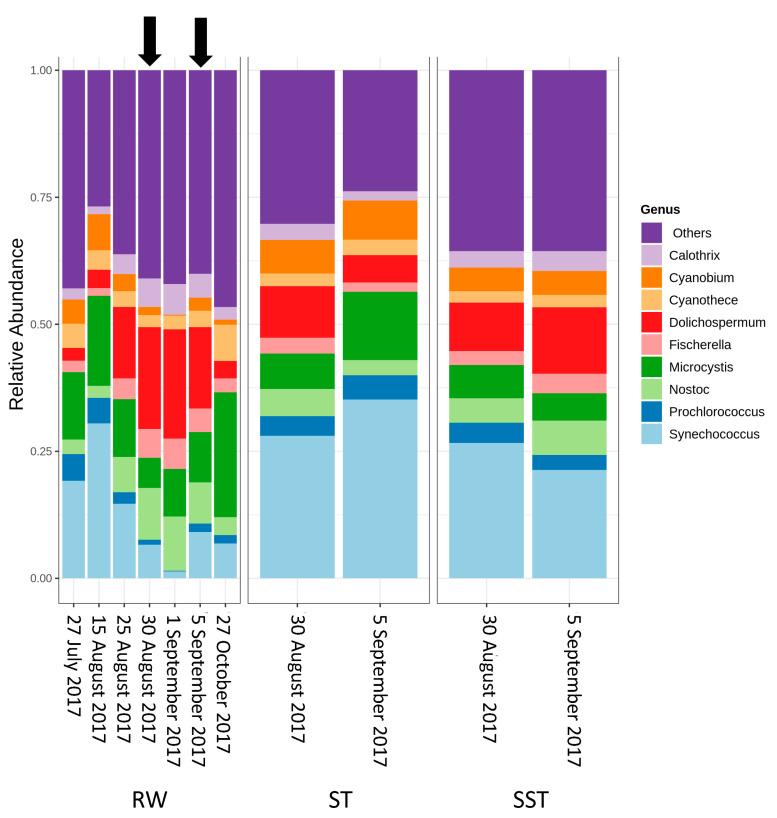
Cyanobacterial community at the genus level in raw water (RW) (27 July, 15, 25, and 30 August, 1 and 5 September, and 27 October 2017), in the sludge holding tank (ST), and in sludge holding tank supernatant (SST) (30 August, 5 September 2017). The black arrows show the corresponding dates for the ST and SST samples.

At the genus level within Cyanobacteria in RW, *Synechococcus* and *Microcystis* were predominant on 27 July and 15 August (Figure 5). In late August, the relative abundance of *Synechococcus* and *Microcystis,* declined, while that of *Dolichospermum* and *Nostoc,* increased. The relative abundance of *Dolichospermum* reached its maximum level on 30 August and 1 September (bloom date). After the bloom date (5 September and 27 October), the relative abundance of *Dolichospermum* decreased, while that of *Microcystis* and *Synechococcus* increased, and the diversity of cyanobacterial communities almost returned to pre-bloom conditions (Figure 5). A previous investigation in Missisquoi Bay documented that the relative abundance of *Dolichospermum* and *Microcystis* repeatedly alternated in bloom and non-bloom events [24], while our study showed that *Synechococcus* also shifted from being a highly abundant taxa before and after the bloom to being present with very low abundance during the bloom. 

**Figure 6:**
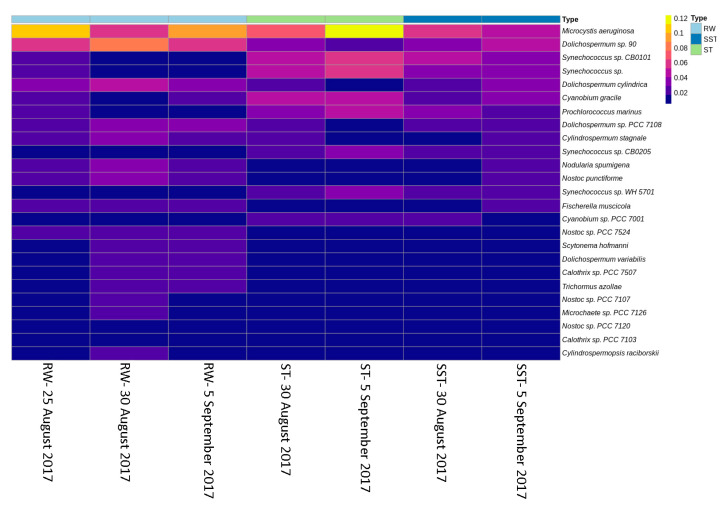
Relative abundance of the top 25 major abundant species in raw water (RW) (25 and 30 August, 5 September 2017), in the sludge holding tank (ST), and in sludge holding tank supernatant (SST) (30 August, 5 September 2017).

**Figure 7:**
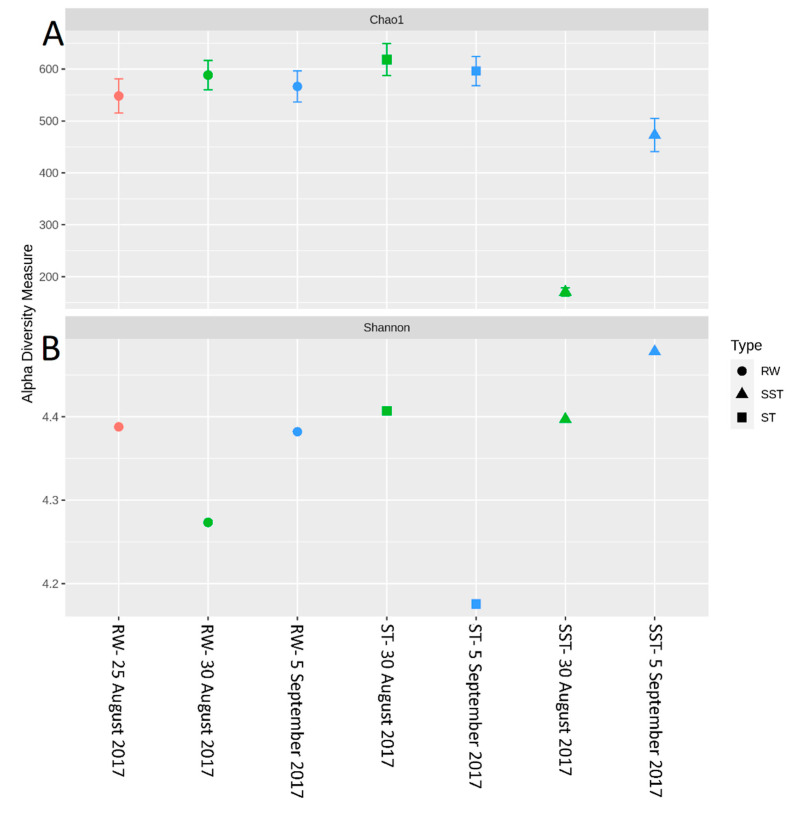
Evaluation of cyanobacterial richness and diversity in raw water (RW) (25 and 30 August and 5 September 2017), in the sludge holding tank (ST), and in sludge holding tank supernatant (SST) on 30 August and 5 September 2017 using (**A**) Chao1 and (**B**) the Shannon index.

The relative abundance of the genera and species changed between RW and ST/SST stages (Figure 5). It is important to note that the structural composition of the sludge communities was not expected to match because sludge is the result of several days of cyanobacterial cell accumulation in the holding tank. For example, when considering samples from 25 August 2017, the sludge holding time was estimated as 13 days, while that of 5 September was 1 day. A comparison between RW (25 and 30 August) and ST (30 August) showed that there was a higher abundance of *Synechococcus* and a lower abundance of *Dolichospermum* in the ST as compared to RW. This trend was also observed on 5 September. On 25 August, the relative abundance of *Microcystis* was lower in the ST in comparison to RW, while the opposite trend was observed on 30 August. On 5 September, the abundance of *Microcystis* was higher in the ST than in the RW. The abundance of *Synechococcus* in SST (30 August) was higher than in RW (25 and 30 August). This trend was also observed on 5 September. The opposite trend of *Synechococcus* was observed within that of *Dolichospermum*. The abundance of *Microcystis* in SST (30 August) was lower than in RW on 25 August but higher than in RW on 30 August. On 5 September, the abundance of *Microcystis* in SST was lower than that in RW. The relative abundance of *Synechococcus* in the ST was higher than that in SST on both sampling dates (30 August and 5 September). On 30 August, the relative abundances of *Microcystis* and *Dolichospermum* were similar in the ST and SST. Interestingly, on 5 September, the abundance of *Microcystis* decreased in SST compared to the ST, while *Dolichospermum* showed the opposite trend (Figure 5). At the species level, similar trends were observed in *M. aeruginosa*, *Dolichospermum sp. 90*, and *Synechococcus sp. CB0101* (Figure 6). Additionally, other genera with lower relative abundance (<6%) were detected in the samples. For example, *Prochlorococcus, Cyanobium, Fischerella, Calothrix*, and *Cyanothece* did not show significant changes between the RW, ST, and SST (Figure 6, Figures S2 and S3). The richness of cyanobacterial species (Chao1 index) remained approximately constant in the RW (578 and 598 on 30 August and 5 September, respectively) and the ST (599 and 620 on 30 August and 5 September, respectively), while it decreased in the SST (275 and 475 on 30 August and 5 September, respectively) (Figure 7a). The difference in richness between RW and ST with respect to SST suggests that the cyanobacterial communities in SST and ST were different but that there were similarities between the ST and RW. 

Between 27 July and 30 August, the Shannon index increased from 4.18 to 4.51 in RW, while it decreased to 4.06 on bloom on 1 September (Figure S4). Our results showed that the diversity decreased during the bloom, in agreement with [24]. The Shannon index in the ST on 30 August (4.4) indicated similar diversity profiles to RW on 25 August (4.39) and 30 August (4.28). Due to 13 days of sludge storage time on 30 August, the diversity in the ST was affected by the bacterial populations in RW in samples on both 25 August and 30 August. The Shannon index in the sludge decreased to 4.16 on 5 September, which is a lower value than the Shannon index in the RW (4.38) on the same date (Figure 7b).

Overall, when relating structural composition of the communities found in raw water (RW), in the sludge holding tank (ST), and in sludge holding tank supernatant (SST), several factors should be considered:

- As expected, changes in composition of RW communities were observed and affected the microbial populations in the ST. Since sludge accumulates over the period of time (1–13 days in this case), the ST profile is expected to reflect accumulative diversity considering both the relative abundance and biomass. Furthermore, the efficacy of coagulation and settling is species-dependent, as shown by [3,17]. Previous investigations also demonstrated that 96–100% of *Dolichospermum* and *Microcystis* cells were more likely to be captured by the clarifier [3,8], and that the coagulation efficiency for these genera was twice the value observed for *Synechococcus* [41].
- The communities found in the ST and SST showed different trends at the phylum and genus level as shown by the Shannon index (Figure S5, Figure 4 and Figure 5). In fact, at the phylum level, Cyanobacteria was selectively removed and retained within sludge (Figure 4). The cyanobacterial community distribution in the supernatant reflects the incoming sludge and the subsequent buoyancy of the community in the sludge. Storage in the holding tank of the sludge may cause cell breakage, leading to vesicle damage [42] and interruption of buoyancy regulation [43]. This would affect the profile of the supernatant in our work. The increase in cyanobacterial richness in SST on 5 September might be due to the longer sludge storage time in the 30 August sample (13 days) compared to that of 5 September (1 day), providing more time for cell damage.
- Cell survival, re-growth, and damage might have occurred in ST during sludge storage. The longer storage of the sludge might have led to cell lysis in the sludge. These phenomena were documented in several studies for various dominant genera including the most dominant genera in this study (*Microcystis* and *Dolichospermum*) after 2 days of sludge storage [4,15,16,44]. Furthermore, trichome damage of *Dolichospermum* due to the treatment stress has been already reported [7]. However, there are no data on the fate of *Synechococcus* in stored sludge.

Understanding the community structure and dynamics in the ST and SST is important for quantitative cyanobacterial risk assessment. Water operators need to be able to predict the exchanges between the sludge and the supernatant. Supernatant (SST) can be discharged into water resources or recycled to the head of the WTP and could constitute a risk for the water intake or an additional burden on the plant treatment processes. Sludge (ST) can be disposed of in wastewater collectors, processed as sludge in lagoons or sludge facilities, or land-applied.

**Figure 8:**
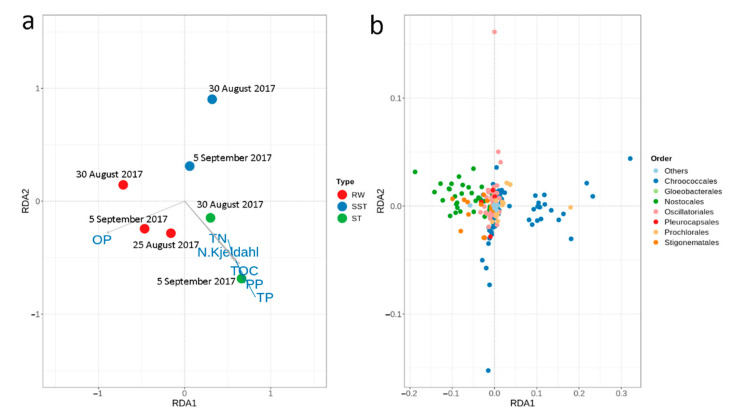
Redundancy analysis (RDA) of cyanobacterial communities with respect to nutrient parameters in (**a**) raw water (RW), in the sludge holding tank (ST), and in sludge holding tank supernatant (SST); (**b**) cyanobacterial distribution at the order level. RDA1: 65.6%, RDA2: 8.7%. Only significant parameters (*p* < 0.05) are shown.

Other studies have shown that environmental conditions can impact sludge communities [16,19]. Redundancy analysis (RDA) analyses were performed to evaluate the relationship between nutrients (Table S1) and cyanobacterial communities. Orthophosphate (OP), total nitrogen (TN, sum of Kjeldahl nitrogen (N- Kjeldahl), organic nitrogen, nitrite, and nitrate), N-Kjeldahl, total phosphorous (TP), particulate phosphorous (PP), and total organic carbon (TOC) exerted significant effects (*p* < 0.05) on community profiles in different ways (Figure 8). A clear correlation was observed between OP in RW, with *Nostocales* (reported to have a 4.5 times higher relative abundance of genes related to phosphorous metabolism than *Chroococcales*) found at low concentrations of phosphorous [45]. Other studies have demonstrated that higher concentrations of nitrogen, phosphorous, and carbon resulted in better conditions for bacterial communities and led to an increase in microbial growth [33,34,46]. In our study, RDA analyses showed that OP was more available in RW than in the ST and SST and that TN, N- Kjeldahl, TP, PP, and TOC had a strong impact on the cyanobacterial population in the ST, which mostly contained *Chroococcales*. None of these nutrient parameters seemed to affect the SST. This is in accordance with our previous observations on the different patterns of cell accumulation in SST. However, it must be noted that the mass of nutrients measured in sludge (2.0–32.8 mg/L of TN and 0.48–5.9 mg/L of TP), was not associated with cyanobacterial cell-bound nutrients nor with dissolved nutrients. Using reference values for cell nutrient content, cell-bound nutrients consist of less than 0.8% nitrogen and 0.4% phosphorous [47]. The persistence of *Chroococcales* in the sludge environment could be the result of the high environmental resistance and the ability to thrive in the presence of elevated levels of nutrients (Figure 8). 

### 2.3. Comparison between Shotgun Metagenomic Sequencing and Taxonomic Cell Counts

The observed genera from microscopic cell counts do not completely match the high-throughput sequencing results, as *Aphanothece*, *Chroococcus*, *Aphanocapsa* and *Coelosphaerium* were not detected by shotgun metagenomics. In RW, *M. aeruginosa, D. circinale, D. spiroides, A. gracile,* and *P. mucicola* were detected through both taxonomic cell counts and metagenomics (data not shown). In contrast, *A. clathrata brevis, C. minimus*, and *C. kuetzingianum* were detected only by taxonomic cell counts and not by metagenomics (data not shown). In ST, only *M. aeruginosa, D. spiroides*, and *D. circinale* were detected by both approaches, while *A. clathrata brevis, C. minimus, P. mucicola, A. delicatissima A. flos-aquae*, and *C. kuetzingianum* were only detected in taxonomic cell counts (data not shown). Overall, 76% and 88% of the detected species by metagenomics were not detected by taxonomic cell counts in RW and ST, respectively (Figure 1 and Figure 6). As recently discussed [48], taxonomic cell counts and high-throughput sequencing can yield different community profiles because of the limitations inherent to each of these methods. Physical and chemical stress in WTPs may cause damaged cells and affect taxonomic cell counts [7], while DNA can be extracted from lysed and dead cells and provide metagenomics shotgun reads [49]. Despite the advantages of taxonomic cell counts, measurement bias such as misidentification of morphologically similar species, the impact of the conservation agent on biovolume, and the complexity of counting species in aggregates should be considered [50,51,52]. In sludge samples, the presence of debris, sediments, and a high number of cells might increase the probability of cross interferences. New metagenomic approaches based on direct cloning and shotgun sequencing of environmental DNA represent a powerful tool for species classification and the evaluation of community dynamics through water treatment processes. However, use of metagenomics also represents some challenges such as: (1) an inadequate recovery rate of DNA [53]; (2) contamination of DNA during extraction [54,55]; and (3) a lack of a standard identification pipeline that includes all species [56].

## 3. Conclusions

- Bacterial communities shifted before and after the cyanobacterial bloom. Proteobacteria was the predominant phylum in RW before the bloom. Levels of Cyanobacteria and Bacteroidetes progressively increased to reach their greatest abundance on the bloom date. This high abundance of Bacteroidetes was associated with nutrient-rich conditions which occurred during the cyanobacterial bloom. After the bloom, bacterial communities returned to almost the same composition as prior to the bloom.
- Conventional treatment eliminated 92–97% of the cyanobacterial cells, as revealed by cell counts. Overall, 96% of *Microcystis* and *Dolichospermum* were eliminated by this process. At first glance, this is an effective approach to controlling the cyanobacterial flux. However, coagulation leads to accumulation of cyanobacterial cells in the sludge. Even a low cell number in intake water (3.9 × 10^4^ cells/mL) led to 31 times as much cell accumulation in the sludge.
- Selective removal of cyanobacteria at the genus and species levels by coagulation/sedimentation has been highlighted by both metagenomic shotgun sequencing and taxonomic cell counts. Sludge (ST) cyanobacterial composition differs from RW if only samples from the same day are considered. Sludge diversity reflects both selective removal by coagulation/sedimentation and the accumulation of captured cells over a period of time as determined by sludge age.
- Monitoring strategies focusing on sporadic measurement of the diversity in raw water cannot capture the risk associated with the storage and disposal of the sludge. Sludge community profiles also appear to be a better indicator for evaluating the influx of cyanobacterial communities in WTPs. Indeed, the sludge profile reflects a cumulative community in terms of relative abundance and biomass.
- Bacterial and cyanobacterial communities of sludge in the holding tank (ST) markedly differed from those measured in sludge supernatant (SST). The communities found in the ST and SST showed different trends at the phylum and genus level as shown by the Shannon index. The prediction of cyanobacterial communities in the supernatant remains a challenge as it is often recycled, possibly adding cyanobacteria and cyanotoxins to the intake water.
- Considering environmental parameters monitored, nutrients were the most discriminating factors affecting cyanobacterial communities. Cyanobacterial communities in RW were influenced by OP, while the sludge communities were correlated with TN, N- Kjeldahl, TP, PP, and TOC.
- Storage, management, and disposal of the cyanobacteria-laden sludge are technical and health-related challenges. By adjusting the storage time and adding PAC, risk assessment of supernatant recycling can be applied to minimize the impact of cyanobacteria and cyanotoxin accumulation.

## 4. Materials and Methods

### 4.1. Description of the Studied Water Body and Plant, Including Treatment Schematics

**Figure 9:**
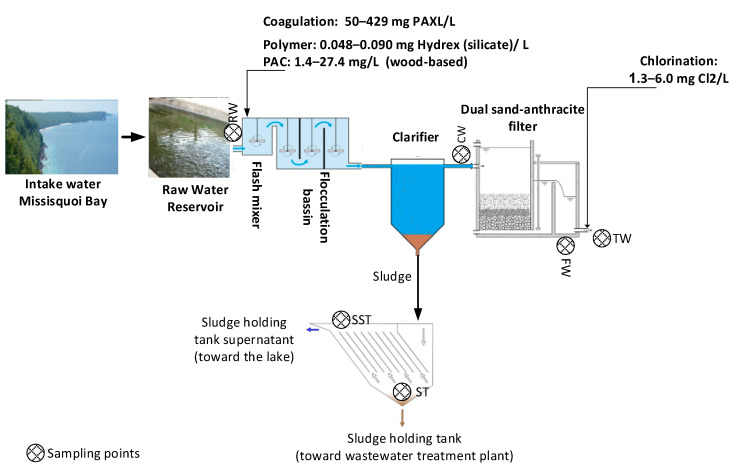
The treatment chain of the WTP and sampling points. The water intake is from Missisquoi Bay. Sampling points are indicated by . RW: raw water; CW: clarified water; FW: filtered water; TW: treated water; ST: sludge holding tank; SST: supernatant of the sludge holding tank.

A plant located on the Canadian side of Missisquoi Bay, Lake Champlain, located South East of Montreal was monitored from 27 July to 27 October 2017. The plant intake water is situated 180 m within the bay. The treatment chain is presented in Figure 9. Briefly, powdered activated carbon (PAC) injection followed by conventional treatment (coagulation, flocculation, sedimentation) and a post-chlorination step are applied as treatment. The clarifier sludge is stored in a sludge holding tank. The supernatant of this sludge is discharged into the lake and the sludge is transferred to the local wastewater treatment plant. 

Overall, seven sampling campaigns were performed on the following dates: 27 July, 15, 25, and 30 August, 1 and 5 September, and 27 October 2017. Specifications of the plant and treatment are summarized in Table S2.

### 4.2. Description of the Studied Water Body and Plant Including Treatment Schematics

Samples were taken following each treatment step from raw water (RW), clarified water (CW), filtered water (FW), treated water (TW), the sludge holding tank (ST), and sludge holding tank supernatant (SST).

Autoclaved 1-L polypropylene bottles, 5-L polypropylene containers, and 40-mL glass vials were used for each sampling point. Before sampling, all containers and vials were rinsed with the water from the sampling point. The 40-mL vials were used for taxonomic cell counts. The taxonomic samples were preserved with Lugol’s iodine. Subsamples were taken for metagenomics from the 1-L bottles; the 5-L containers were used for cell-bound and dissolved microcystins (MCs) and nutrient samples.

Genomic subsamples were prepared by sample filtration via 0.2-μm polyethersulfone hydrophilic membranes (Millipore Sigma, Oakville, ON). Membranes were stored in the sterile Falcon tube at −80 °C. Cell-bound and dissolved microcystin subsamples were prepared by sample filtration using pre-weighted 0.45-μm GHP membranes (Pall, Mississauga, ON). The filters were kept in the petri dish and the filtrate was kept in 125-mL graduated polyethylene terephthalate glycol (PETG) amber bottles (Thermo Fisher, Mississauga, ON). Subsamples for total nitrogen (TN), total phosphorous (TP), and total organic carbon (TOC) were aliquoted directly. Dissolved nitrogen (DN), Kjeldahl nitrogen (N- Kjeldahl), ammonium nitrogen (NH_4_), nitrite/nitrate (NO_2_/NO_3_) and dissolved phosphorous (DP) subsamples were filtered on 0.45-μm membranes (Millipore Sigma, Oakville, ON). 

Genomic subsamples were taken in triplicate, while MC and nutrient samples were taken in duplicate. MC, N- Kjeldahl, and NO_2_/NO_3_ subsamples were frozen at −25 °C. TN, TP, and TOC samples were stored at 4 °C. Taxonomic cell count samples were stored in a dark place at ambient temperature.

### 4.3. Description of Analytical Methods

#### 4.3.1. Taxonomic Cell Counts

Taxonomic cell counts were performed by an inverted microscope in a Sedgwick-Rafter chamber at magnifications of 10 and 40× according to [57,58,59]. 

#### 4.3.2. Microcystin Analysis

Total microcystins (MCs) were analyzed by on-line solid-phase extraction ultra-high-performance liquid chromatography coupled to tandem mass spectrometry (on-line SPE-UHPLC-MS/MS). Firstly, samples were oxidized by potassium permanganate and sodium (meta) periodate (Sigma Aldrich, Oakville, ON, Canada). Secondly, samples were quenched by a 4M sodium bisulfite solution (Sigma Aldrich, Oakville, ON, Canada). Thirdly, the standard solutions of 4-phenylbutyric acid (50 ng/L) (Sigma Aldrich, Oakville, ON, Canada) and erythro-2-Methyl-3-methoxy-4-phenylbutyric acid (D3-MMPB, 10 ng/L) (Wako Pure Chemicals Industries, Ltd., Osaka, Japan) were added to the samples. Fourthly, 10 mL of solution were filtered using 0.22-μm nylon filters (Sterlitech Corporation, Kent, WA, USA). Aliquots were taken for analysis using the Thermo EQUAN™ interface (Thermo Fischer Scientific, Waltham, MA, USA). The “in-loop” injection was controlled by an HTC Thermopal autosampler (CTC analytics, Zwingen, Switzerland). Then, samples were loaded into a Thermo Hypersil Gold aQ C18 (on-line SPE) column (20 mm × 2.1 mm, 12 μm). Separation of toxins was performed on a Thermo Hypersil Gold C18 column (100 mm × 2.1 mm, 1.9 µm). MS/MS detection was performed by thermo TSQ QUANTIVA triple quadrupole mass spectrometer (Thermo Fischer Scientific) following UHPLC. Water, methanol, and acetonitrile for HPLC were purchased from Fisher Scientific (Whitby, ON, Canada) and formic acid (>95%), potassium carbonate, ammonium hydroxide (28–30% NH3), and ammonium acetate (≥99.0%) were obtained from Sigma Aldrich (Oakville, ON, Canada). More details are provided by Munoz et al. [60] and Roy-Lachapelle et al. [61].

#### 4.3.3. Nutrient Analysis

Nitrogen, nitrite, nitrate, and ammonium nitrogen were analyzed by the colorimetric technique based on EPA 350.1 and 353.2 standard methods [62,63]. Phosphorous and phosphate were measured by the colorimetric technique based on EPA 365.1 and 365.3 methods [64]. TOC was analyzed by Sievers InnovOX Laboratory TOC analyzer (USA) based on USEPA 415.1 method [65].

### 4.4. DNA Extraction and Metagenomics Preparation

The extraction of total nucleic acid from frozen filters was performed with the RNeasy PowerWater Kit (Qiagen, Toronto, ON, USA) with slight modifications. To avoid formation of disulfide bonds protein residuals, dithiothreitol (DTT) was spiked into the pre-warmed (55 °C) PM1 buffer. Since substantial biomass remained on the surface of the membrane after the bead-beating step, the filters were transferred alongside the supernatant and were incubated with the IRS solution. Total nucleic acids were eluted with 60 µL of nuclease-free water. Half of the sample was treated with RNase If (New England Biolabs, Whitby, ON) to remove the RNA. The resulting DNA was purified using the Genomic DNA Clean & Concentrator TM-10 (Zyimo Research Corporation, Irvine, CA, USA), following the instructions of the manufacturer. 

For the sludge samples, the RNeasy PowerSoil Total RNA Kit (Qiagen, Toronto, ON, Canada) was used with two modifications to improve the recovery of DNA. The modifications consisted of (1) a centrifugation step for 5 min at 13,000 rpm at 4 °C to separate the water from the sludge; and (2) incubation at −20 °C for 60 min after the addition of solution SR4 to precipitate nucleic acids. DNA was eluted using the PowerSoil DNA Elution Kit (Qiagen, Toronto, ON, Canada), following the instructions of the manufacturer. DNA quantification was done by Qubit V2.0 fluorometer (Life Technologies, Burlington, ON, Canada). The metagenomic libraries (Roche 454 FLX instrumentation with Titanium chemistry) were sent to McGill University and Genome Quebec Innovation Centre for sequencing. Ninety-six libraries were then pooled together and sequenced on a NovaSeq 6000 S4 with paired end of 150 bp and an insert of 360 bp.

Metagenomic analysis were performed on all RW, ST, and SST samples collected on 5 August and 5 September. Community dynamics was assessed using shotgun metagenomics levels of phylum, order, genus and species. The number of reads for taxonomic data was normalized by relative abundance. 

### 4.5. Bioinformatics Analysis

DNA libraries were sequenced on the Illumina NovaSeq 6000 platform using S4 flow cells. Paired-end raw reads of 150 base pairs (bp) were further analyzed using a home-made bioinformatics pipeline. Firstly, quality trimming of raw reads was performed by the SolexaQA v3.1.7.1 program with default settings [66]. Trimmed reads shorter than 75 nt were removed for further analysis. Artificial duplicate removal was performed using an in-house script based on the screening of identical leading 20 bp. From the trimmed high-quality reads, gene fragments were predicted using FragGeneScan-Plus v3.0 [67]. Cd-hit v4.8.1 was applied to cluster predicted protein fragments at a 90% similarity [68]. One representative of each cluster was used for a similarity search on the M5nr database (https://github.com/MG-RAST/myM5NR) using the Diamond engine [69]. For assessment of taxonomic affiliation of gene fragments encoding proteins, we took into account best hits (minimal e-value of 1 × 10^−5^) combined with a last common ancestor approach. 

### 4.6. Statistical Analysis

Statistical analysis was performed by R (3.6.2). Bacterial communities at the phylum, order, and genus level were analyzed by phyloseq (1.28.0) [70]. Taxonomic data was normalized by centered log-ratio transformation using easyCODA (0.31.1) [71]. Then, the cyanobacteria species community was analyzed based on the first 25 most frequent species by pheatmap (1.0.12) (https://CRAN.R-project.org/package=pheatmap). The richness index was analyzed by phyloseq’s estimate_richness function. For visualization of the species community and diversity variation, heat trees were illustrated using the metacoder (0.3.3) [72]. Beta-diversity was performed by vegan package (2.5–6) (https://CRAN.R-project.org/package=vegan). Similarity matrices were calculated according to Euclidean distance. A redundancy analysis (RDA) was performed to evaluate the impact of constrained variables on sampling points at >95% significance. The homogeneity of variances was validated before the model implementation. A model was defined by the ordistep function [73] to illustrate the impact of nutrient parameters on the distribution of cyanobacterial communities in the RW, ST, and SST at the order level. The Envfit function was used to find similar scores and to scale the fitted vectors of variables based on the correlations. The permutation test (>95% significance) was applied to select significant variables.

## Data Availability

Data is contained within the article and supplementary material.

## Supplementary Materials

The following are available online at https://www.mdpi.com/2072-6651/13/1/25/s1. Figure S1. Principal component analysis (PCA) of nutrient parameters’ impact on (**a**) sampling dates, and (**b**) bacterial diversity (phylum-level) in raw water (RW) on 27 July, 15, 25, and 30 August, 1 and 5 September, and 27 October 2017. Only the significant parameters are shown (*p* < 0.05). Figure S2. Relative abundance of the top 25 major abundant genera in RW. Samples taken on 27 July, 15, 25, and 30 August, 1 and 5 September, and 27 October 2017. Figure S3. Relative abundance of the top 25 major abundant genera in the sludge holding tank (ST) and sludge holding tank supernatant (SST). Samples taken on 30 August and 5 September 2017. Figure S4. Evaluation of the cyanobacterial diversity in raw water (RW) on 27 July, 15, 25, and 30 August, 1 and 5 September, and 27 October 2017 using the Shannon index. Figure S5. Bacterial diversity in the raw water (RW) on 25 and 30 August and 5 September 2017, and in the sludge holding tank (ST) and sludge holding tank supernatant (SST) on 30 August and 5 September 2017. Table S1. Concentration of nutrients in raw water (RW), in the sludge holding tank (ST), and in sludge tank supernatant (SST) on 27 July, 15, 25, and 30 August, 1 and 5 September, and 27 October 2017. Table S2. Concentrations of cell-bound and dissolved microcystins (MCs) in the RW (raw water), sludge holding tank (ST), and sludge holding tank supernatant (SST). Table S3. Water characteristics of the studied plant in Missisquoi Bay during the sampling campaign from July to October 2017.
