# Supplementary material for: Can Cyanobacterial Diversity in the Source Predict the Diversity in Sludge and the Risk of Toxin Release in a Drinking Water Treatment Plant?
Source: Toxins (Basel). 2021 Jan 1;13(1):25. doi: 10.3390/toxins13010025 (PMC7823770; doi:10.3390/toxins13010025)
Supplement: Supplementary file 1 [file toxins-13-00025-s001.pdf]

Can Cyanobacterial Diversity in the Source Predict the Diversity in Sludge and the Risk of Toxin Release in a Drinking Water Treatment Plant?

Farhad Jalili, Hana Trigui, Juan Francisco Guerra Maldonado, Sarah Dorner, Arash Zamyadi, B. Jesse Shapiro, Yves Terrat, Nathalie Fortin, Sébastien Sauvé and Michèle Prévost

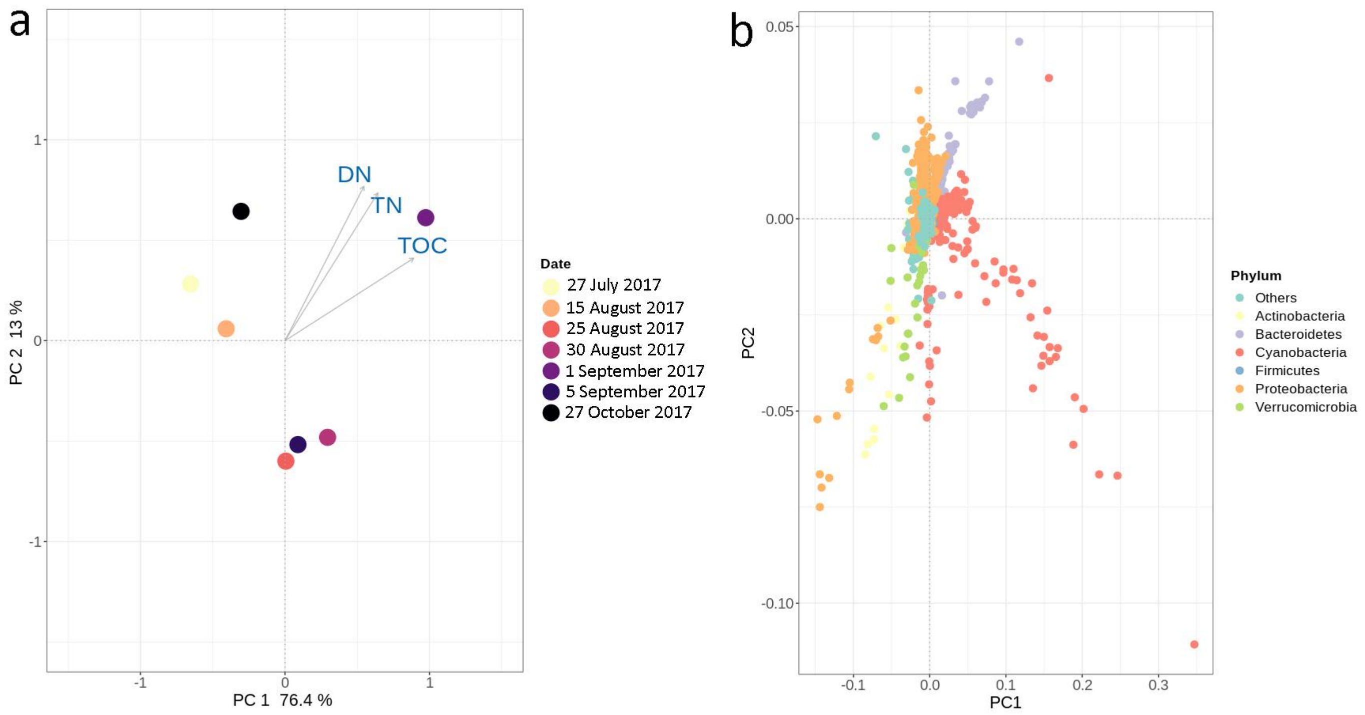

Figure S1. Principle components analysis (PCA) of nutrient parameters' impact on **a**) sampling dates, **b**) bacterial diversity (phylum level) in raw water (RW) on 27 July, 15, 25, 30 August, 1, 5 September and 27 October 2017. Only the significant parameters were shown ($p < 0.05$).

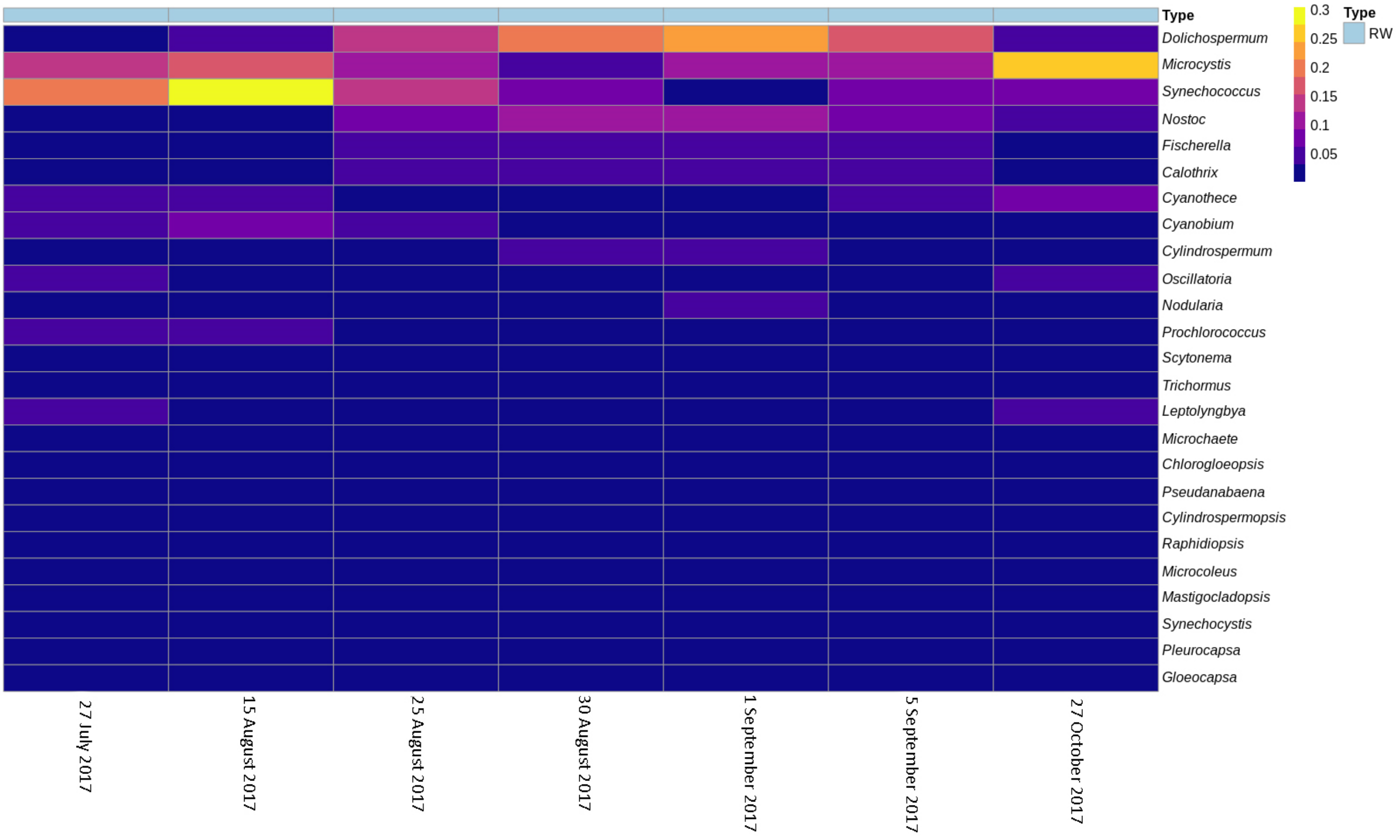

Figure S2. Relative abundance of the top 25 major abundant genera in the raw water (RW). Samples taken on 27 July, 15, 25, 30 August, 1, 5 September and 27 October 2017.

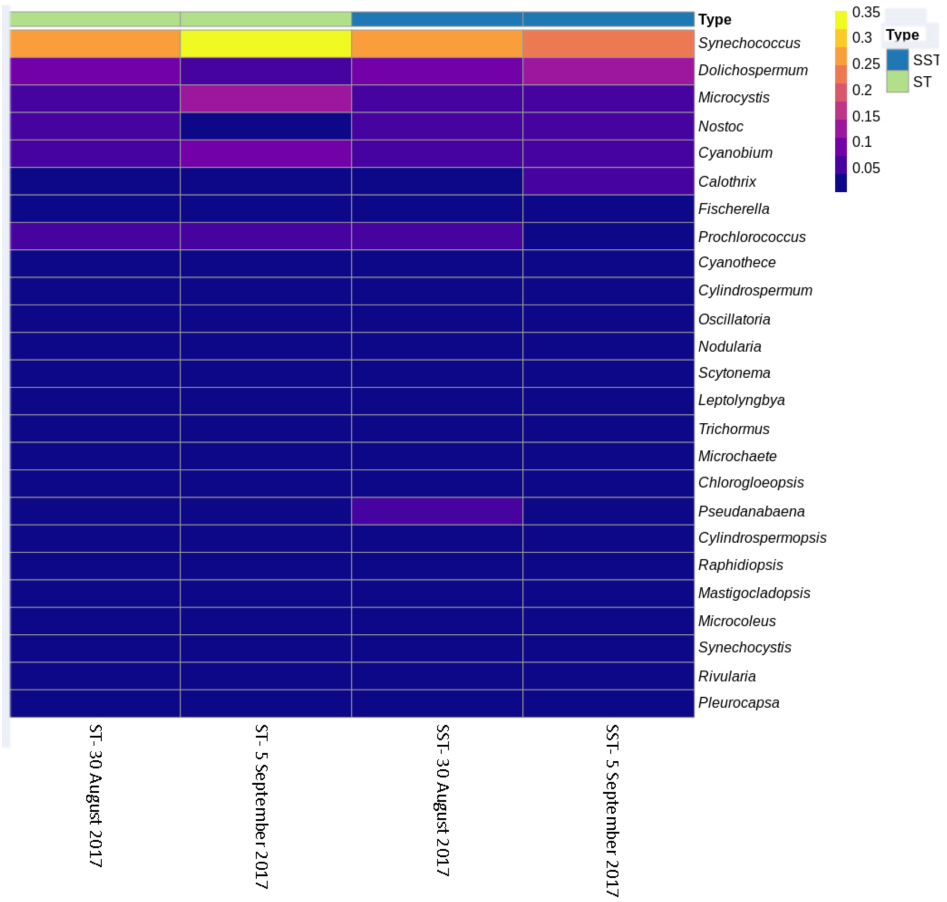

Figure S3. Relative abundance of the top 25 major abundant genera in sludge holding tank (ST) and sludge holding tank supernatant (SST). Samples taken on 30 August and 5 September 2017.

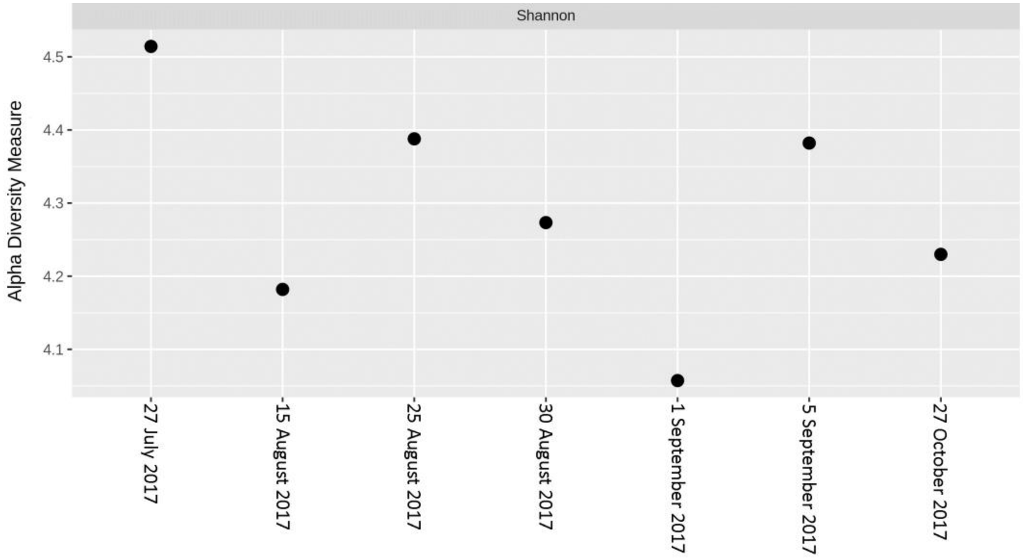

Figure S4. Evaluation of the cyanobacterial diversity in raw water (RW) on 27 July, 15, 25, 30 August, 1, 5 September and 27 October 2017 using Shannon index.

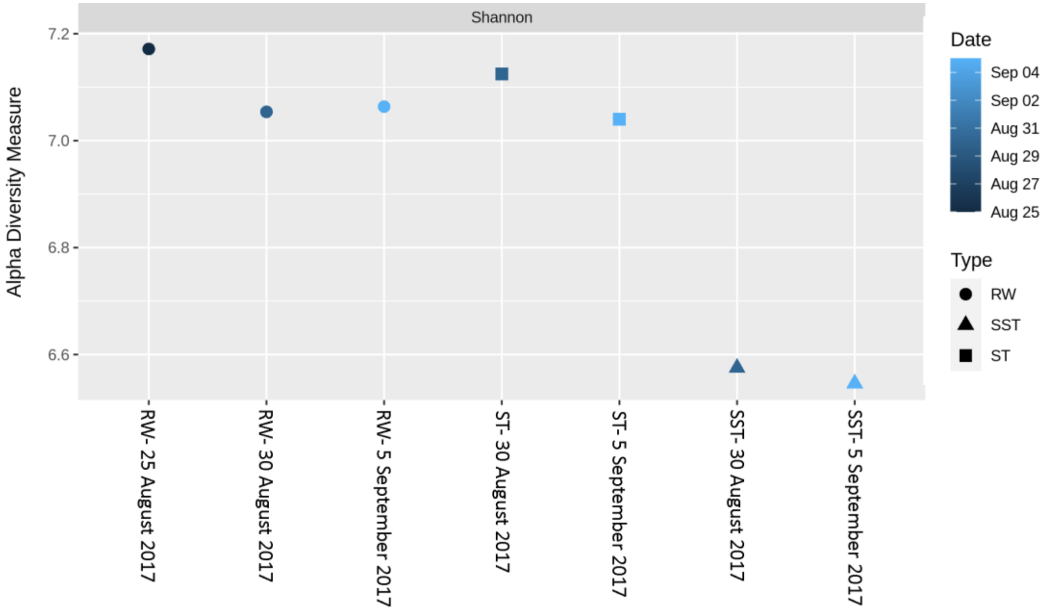

Figure S5. Bacterial diversity in the raw water (RW) on 25, 30 August and 5 September 2017, sludge holding tank (ST) and sludge holding tank supernatant (SST) on 30 August and 5 September 2017.

Table S1. Concentration of nutrients in raw water (RW), sludge holding tank (ST) and sludge tank supernatant (SST) on 27 July, 15, 25, 30 August, 1, 5 September and 27 October 2017. NS: Sample not taken.

Date	TOC (mg C/L)			TN (mg N/L)			DN (mg N/L)			N-Kjeldahl (mg N/L)			NH4 (mg N/L)			DON (mg N/L)			NO2- NO3 (mg N/L)		
	RW	ST	SST	RW	ST	SST	RW	ST	SST	RW	ST	SST	RW	ST	SST	RW	ST	SST	RW	ST	SST
27 July 2017	5.30	13.85	NS	0.87	2.19	NS	0.78	0.76	NS	0.60	1.80	NS	0.17	0.13	NS	0.40	0.30	NS	0.25	0.37	NS
15 August 2017	5.30	220.00	2.70	0.96	1.98	0.94	0.84	0.83	0.80	0.60	1.40	0.40	0.07	0.06	0.05	0.40	0.20	0.20	0.41	0.56	0.56
25 August 2017	5.30	290.00	2.90	0.64	3.09	0.47	0.58	0.54	0.43	0.60	3.00	0.30	0.09	0.11	0.06	0.40	0.30	0.20	0.08	0.10	0.13
30 August 2017	6.10	1390.00	2.60	0.97	29.21	0.53	0.68	1.15	0.46	0.80	29.00	0.40	0.12	0.44	0.09	0.40	0.50	0.20	0.13	0.18	0.17
1 September 2017	8.60	1240.00	NS	1.61	32.77	NS	1.25	1.86	NS	0.01	32.60	NS	0.27	0.46	NS	0.80	1.20	NS	0.23	0.16	NS
5 September 2017	5.80	430.00	2.70	0.84	5.44	0.52	0.68	0.56	0.51	0.70	5.20	0.30	0.08	0.06	0.08	0.40	0.30	0.20	0.19	0.21	0.21
27 October 2017	5.60	2.40	NS	1.12	2.27	NS	0.82	0.72	NS	0.80	1.90	NS	0.07	0.05	NS	0.40	0.30	NS	0.32	0.37	NS

Date	TP (µg P/L)			DP (µg P/L)			OP (µg P/L)			PP (µg P/L)			DOP (µg P/L)		
	RW	ST	SST	RW	ST	SST	RW	ST	SST	RW	ST	SST	RW	ST	SST
27 July 2017	54.51	323.41	NS	31.54	2.34	NS	18.00	4.00	NS	23.00	321.00	NS	14.00	2.00	NS
15 August 2017	93.32	476.00	18.00	47.70	3.00	4.00	35.00	3.00	2.00	46.00	473.00	14.00	13.00	2.00	4.00
25 August 2017	60.64	948.00	36.00	27.50	8.00	11.00	21.00	4.00	6.00	27.00	940.00	25.00	12.00	4.00	4.00
30 August 2017	78.35	5277.00	21.82	27.49	29.00	4.30	14.00	4.00	3.00	51.00	5248.00	18.00	13.00	25.00	2.00
1 September 2017	50.72	5857.00	NS	23.73	60.00	NS	8.00	25.00	NS	27.00	5797.00	NS	16.00	60.00	NS
5 September 2017	85.89	1176.00	17.65	42.56	7.00	7.12	24.00	3.00	3.00	43.00	1169.00	11.00	19.00	4.00	4.00
27 October 2017	119.01	554.00	NS	42.29	3.00	NS	29.00	2.00	NS	77.00	551.00	NS	14.00	3.00	NS

Table S2. Concentrations of cell-bound and dissolved microcystins (MCs) in the RW (raw water), (ST) sludge holding tank and (SST) sludge holding tank. DL: below detection limit. NS: sample mot taken.

Date	RW		ST		SST	
	**Cell-bound MCs (ng/L)**	**Dissolved MCs (ng/L)**	**Cell-bound MCs (ng/L)**	**Dissolved MCs (ng/L)**	**Cell-bound MCs (ng/L)**	**Dissolved MCs (ng/L)**
27 July 2017	2.9	65.7	5.1	54.8	NS	
15 August 2017	5.8	81.5	11.6	77.6	3.3	75.6
25 August 2017	6.0	89.6	36.1	74.2	13.0	86.7
30 August 2017	9.0	64.0	38.9	52.6	44.7	71.3
1 September 2017	68.2	191.9	158.9	121.7	NS	
5 September 2017	DL	DL	12.7	69.2	36.9	91.2
27 October 2017	20.7	121.9	52.4	116.7	NS	

Table S3. Water characteristics of the studied plant in Missisquoi Bay during the sampling campaign from July to October 2017.

Treatment Step	Parameters	July	August	September	October	Specifications
Raw water (RW)	Turbidity (NTU)	2.4–93.3	0.14–102.5	2.7–79.8	1.5–153	-
	pH	6.0–8.2	5.6–8.2	6.4–9.1	5.9–8.7	-
Clarifier (CW)	Turbidity (NTU)	0.29–20	0.01–10.23	0.01–20.1	0.24–21.1	Effective clarifier depth: 4.90 m, Max. sludge bed: 2.95 m, Hydraulic retention time: 1 h, Solid retention time: 48 h
	pH	6.1–7.3	6.66–7.11	6.20–7.00	6.54–7.05	
Dual sand-antrachite filter (FW)	Turbidity (NTU)	0.16–0.4	0.15–0.4	0.12–0.4	0.1–0.5	Retention time: 2 h
Treated water (TW)	Turbidity (NTU)	0.24–0.59	0.22–0.50	0.20–0.45	0.15–0.41	-
	pH	6.51–8.02	7.04–8.00	6.95–8.74	7.10–8.14	-
